# Zakat, Non-state Welfare Provision and Redistribution in Times of Crisis: Evidence from the Covid-19 Pandemic

**DOI:** 10.1007/s12116-024-09447-x

**Published:** 2024-10-26

**Authors:** Max Gallien, Umair Javed, Vanessa van den Boogaard

**Affiliations:** 1 Institute of Development Studies, Brighton, UK; 2 Lahore University of Management Sciences, Lahore, Pakistan; 3 University of Toronto, Toronto, Canada

**Keywords:** Zakat, Non-state social welfare provision, Social protection, Covid-19

## Abstract

Around the world, the Covid-19 pandemic drew attention to state social protection and its limitations. Less attention has been paid to what is likely the world’s largest system of predominantly non-state welfare provision: zakat, an annual Islamic obligatory payment of a percentage of productive wealth to the poor and other eligible recipients. We explore how states and citizens engage with zakat during crises through a case study of the Covid-19 pandemic in Pakistan, Egypt, and Morocco, drawing on novel and nationally representative survey data of 5484 respondents across the three countries. While we may expect that citizens may be less motivated to pay zakat in times of personal economic hardship, we find that a large majority of the general population and of zakat contributors perceives zakat as particularly important in the Covid context. We show that while zakat may play an important role in non-state social welfare provision supplementing state social protection and redistribution in times of crisis, state attempts to harness it are often ineffective. However, while we find that higher income individuals are more likely to pay zakat, even only among those that are eligible, there are potentially negative equity impacts given the flat rate at which it is levied and the fact that people tend to give through personal networks.

## Introduction

Following the Covid-19 pandemic, there has been considerable attention to state-led social protection programs and their limitations. While many governments introduced or expanded social protection programs,^1^ research has highlighted patterns of exclusion in state relief programs for vulnerable groups, including informal and migrant workers (van den Boogaard et al. 2024; Gallien and van den Boogaard 2021; Pande 2020; Raju et al. 2021).^2^ More fundamentally, financing constraints and public debt crises imply limits to the reach of state crisis responses, particularly in lower income contexts. With the recognition of these limitations, the role of non-state social welfare provision in responding to the crisis is important to consider.

There is a significant literature on non-state social welfare provision (Cammett and Issar 2010, Cammett 2014, Brooke 2019, Medani 2021) and some analyses of the ways in which non-state actors have played a “large role in supporting social welfare and providing public goods either in parallel to or in conjunction with the state” in response to the Covid-19 pandemic (Gallien and van den Boogaard 2021; see also Leach et al. 2021; Osuteye et al. 2020; van den Boogaard et al. 2024). Yet there has been comparatively little analysis about the role and implications of zakat during the Covid-19 pandemic and other crises, despite it likely being the world’s largest system of predominantly (though not exclusively) non-state welfare provision. One of the five pillars of Islam, zakat represents an annual obligation to pay 2.5% of total productive wealth above an eligibility threshold, the *nisab*, to a set of eligible recipients, including the poor.^3^ Accordingly, it is often described as a form of wealth redistribution and welfare provision (Ahmad 1991). We explore citizen perceptions of the role of zakat in responding to the Covid crisis in three Muslim-majority countries—Pakistan, Egypt, and Morocco—relying on novel and nationally representative survey data collected in 2020 with 5484 Sunni Muslim respondents across the three countries.

We first investigate citizen experiences with and perceptions of non-state social welfare, in the form of both zakat and charitable giving (*sadaqah*). On the one hand, financial hardship may limit non-state social welfare provision and peoples’ feeling of obligation to pay. On the other hand, increased need and feelings of social solidarity—and potentially a recognition of the limitations of state social protection—may reinforce the importance of social protection beyond the state. While we may expect that citizens may be less motivated to pay zakat in times of personal economic hardship, we find that a large majority of the general population and of those eligible to make zakat payments perceive zakat as important in the Covid context. Across our case studies, a higher proportion of respondents believe that zakat is relatively more important than taxation in responding to the crisis, despite tax being the fiscal instrument most associated with welfare responses. At the same time, people also report giving more in *sadaqah* contributions in the pandemic relative to the previous year, indicating that social solidarity expanded during the crisis in these countries, despite economic hardship. This suggests that rather than limiting their zakat and charity contributions in response to personal economic hardships, citizens feel a greater sense of the importance of redistribution — outside of state structures.

Second, with this understanding that non-state social welfare may play an important role in supplementing state social protection and redistribution in times of crisis, we explore the potential distributional impacts of zakat and sadaqah. This is particularly important in the context of increasing poverty and inequality resulting from the Covid pandemic (Mahler et al. 2022). We find that, in line with expectations, higher income groups are more likely to report paying both zakat and sadaqah, even when controlling for zakat eligibility. While we are unable to assess the overall progressivity (whether richer individuals pay more as a share of their wealth), the fact that zakat is levied at a flat rate indicates that it is likely to be regressive among those that make any payment. We further consider the implications of the way that zakat is distributed—predominately through personal and social networks. On the one hand, individuals may have an informational advantage over the state in identifying those in need; on the other hand, personalized giving through social networks may risk reproducing social stratifications and exclusions. We identify the distributional impacts of zakat as an important area for further research.

Overall, we show that zakat has represented an important source of social protection and welfare provision in at least some Muslim-majority countries during the Covid pandemic. Our findings are in line with evidence of the power and prevalence of bottom-up responses during times of crisis (Dynes and Tierney 1994; Quarantelli 2008; Solnit 2010); that is, rather than expectations that zero-sum mentalities and conflict will emerge in times of crisis, communal solidarities are often strengthened or emerge. Our evidence thus contributes to the literature on social protection and welfare provision and ‘bottom-up initiatives’ in a crisis context. It highlights the importance of incorporating zakat within analyses of social protection in times of crisis.

We build these arguments throughout the rest of the paper. The “Background: Zakat and Non-state Social Protection and Welfare Provision” section provides background on non-state social protection and welfare provision more generally and zakat in particular. The “Case study context” section describes the country case selection and provides background on these states’ role in mobilizing zakat to supplement state social protection during the pandemic. The “Data” section describes the survey data upon which we rely. The “Citizen Perceptions of Zakat and Sadaqah during Crises” section presents data on citizen perspectives of the role of zakat and sadaqah in times of crisis, while the “Potential distributional impacts of zakat” section considers the role of zakat in supplementing state social protection, highlighting both the possibilities and risks of relying on this form of non-state social welfare provision from an equity perspective. The “Conclusions” section discusses the implications of the key findings and identifies areas for future research.

## Background: Zakat and Non-state Social Protection and Welfare Provision

The role of non-state social welfare provision in contributing to development and supplementing weak state capacity has received considerable scholarly attention (Cammett and MacLean 2014b; Kushner and MacLean 2015; MacLean 2017).^4^ In low-income contexts, states often rely on non-state actors to provide relief to vulnerable groups excluded by state social protection (Gerard et al. 2020)—which, given high levels of informal employment in low-income countries, often means the majority of individuals (Gallien and van den Boogaard 2021). This body of work has drawn critical attention to the need to de-centre the state in analysis of public goods provision, in part recognizing that state involvement in financing and delivering public goods and social welfare is a modern concept that does not inherently follow from state authority. A key focus has been on exploring the relationships between the state and non-state social welfare providers, with non-state actors operating “beside”, ‘below”, or “beyond” the state (Bellagamba and Klute 2008).^5^ Across diverse contexts, evidence shows the ways in which non-state social welfare can play a complementary or supplementary role to the state, essentially filling gaps left by weak public finances without necessarily undermining the state or its legitimacy (van den Boogaard 2020; Cammett and MacLean 2014a; Post et al. 2017; Sacks 2012).

Much of this literature has focused on welfare provision by private actors (e.g., Cook 2014; Katusiimeh 2015; Luong 2014; Mizala and Schneider 2014; Post 2014; Pritchett and Viarengo 2015), non-governmental organisations (e.g., Allard 2014; Brass 2014; 2016), or local solidarity networks (e.g., van den Boogaard, Orgeira, and Prichard Forthcoming; van den Boogaard and Santoro 2022; Evans et al. 2020; MacLean 2014). In the context of the Middle East and North Africa, scholarship has further highlighted the ways in which non-state social welfare provision relates to Islamist movements or Islamic charity and its potential to fill gaps left by limited state provision, particularly in times of economic crisis or following conflicts (Clark 2004; Masoud 2014; Cammett and MacLean 2014b; Cammett and Luong 2014; Brooke 2017, 2019; Cammett 2014; Medani 2021). Comparatively little explicit attention has been paid to the ways in which zakat fits within these models of non-state social welfare, and in particular the role of zakat in responding to crises. In the context of the pandemic, existing research either focuses on the potential of zakat payments to aid in the pandemic response (Rabbani et al. 2021) or delineates the efforts made by state zakat funds and organisations to adjust and respond to the pandemic (Swandaru and Abdel 2022).

Zakat is described in the Quran as an entitlement of the poor on the wealth of those who are well off, as well as a way to purify the belongings of the wealthy through almsgiving (Quran, 9: 103). In contrast to *sadaqah,* which describes voluntary charitable payments made with the intention of pleasing god, zakat is conceptualised as an obligation in Islam, rather than merely a good deed.^6^ With the poor and needy explicitly identified among the eight categories of zakat recipients in the Quran,^7^ zakat is often discussed in the context of wealth redistribution (Green 2015), direct transfers to poor populations, poverty alleviation programs, and humanitarian assistance (Stirk 2015). There has been some debate on the role of poverty alleviation in its mandate (Kuran 2003) and how effectively it is employed to that end (Ali and Hatta 2014). Although there is good reason to be skeptical about the accuracy of some estimates, zakat is clearly a substantial part of global social spending: lower estimates put the annual global zakat pool at 200 billion USD— meaning that it may surpass the annual total spending on overseas development assistance by OECD countries.^8^ In many Muslim-majority countries, estimated zakat contributions exceed the amount spent on state social protection programs. In Morocco, for example, even the lowest estimate of yearly zakat contributions are three times the budget of the National Initiative for Human Development, the country’s flagship social assistance program (ESCWA n.d.)

Notably, zakat is organized and managed at different levels and by different actors. People make zakat payments to religious and secular NGOs, hospitals, schools, mosques, and internationally organisations (May 2013; Weiss 2020).^9^ In a majority of Muslim-majority countries, payments are also made to funds organised by centralised or sub-national state bodies. Giving to these funds rather than to other recipients is mostly voluntary, though some — including in Pakistan, Malaysia and Saudi Arabia — mandate and enforce payments from citizens by, for example, withdrawing them from savings accounts and other financial instruments (Migdad 2019, Gallien et al. 2023). While giving to collective funds or organizations may be a particularly visible form of giving in a crisis context, just focusing on these modalities obscures the magnitude of zakat giving. Much zakat — and the majority in the case studies discussed here — is given directly to individual recipients (Gallien et al. 2023).

Given its magnitude and flexibility, zakat has played an important role in previous disaster and emergency relief situations (May 2013). Examples include the role of zakat foundations in providing relief for flood victims in Pakistan in 2010 and in Eastern Malaysia in 2015 (Wahid et al. 2018; Zaenal et al. 2019). The potential for zakat to contribute to managing the social and economic fallout of crises such as the Covid pandemic is hence substantial. To the best of our knowledge, however, the role and dynamics of zakat during crises from the perspective of zakat contributors or the population at large has not been explored. We fill this gap below, documenting state actions, citizen perceptions, and potential distributional impacts of zakat in the context of the Covid-19 pandemic.

## Case Study Context

To explore perceptions of zakat in times of crisis, we consider three country case studies— Egypt, Pakistan, and Morocco—capturing over 15% the global Muslim population across three regions and representing contexts that have largely been unexplored by the emerging literature on zakat and social protection, much of which remains focused on Southeast Asia (Hudaefi et al. 2021; Umar et al. 2021). These countries share important similarities that make them suitable to consider together. Though Pakistan has a lower GDP per capita, they are all lower middle-income, Sunni-majority countries.^10^ All were significantly affected by the economic and public health crises related to the Covid-19 pandemic.

Furthermore, these cases vary across two key dimensions which we consider in our analysis. First, to some degree, jurisprudential differences influence different interpretations and practices of zakat, including with respect to the threshold at which individuals are obligated to pay (the nisab).^11^ In Pakistan, where the Sunni Hanafi jurisprudence is practiced by the majority, the silver nisab is generally used to calculate zakat obligations.^12^ In Egypt and Morocco, in line with other Sunni jurisprudential schools (*maddhabs*), a nisab based on the gold value—and hence a considerably higher threshold—is more common.^13^ This has implications for the extent of zakat eligibility and obligations across countries. To account for these differences, we present the data below disaggregated by both national averages and averages among those who are above the respective threshold.

Second, across the three countries the role of the state in zakat administration differs. Like most Muslim-majority countries, Pakistan and Egypt have state-run zakat funds. In Pakistan, payment into a central fund operated by the Ministry of Religious Affairs is collected from savings accounts and similar financial instruments, and then transferred downward to provincial, district, and local zakat councils. Egypt’s state fund, by contrast, is only made up of voluntary donations, with the state not actively collecting any zakat from bank accounts. In Morocco, like in a small number of other Muslim-majority countries including Tunisia and Turkey, there is no state-run fund, and donations are managed solely by citizens and non-state organisations. The existence of these different forms of state engagement with zakat across our case studies ensure that our analysis is not conditional upon one type of state engagement.

Despite these differences in the state’s role in zakat administration, governments in all three countries played a role in encouraging zakat contributions during the pandemic and by promoting state relief funds as eligible and worthy recipients for zakat contributions, as they often have during other crises. In part, this reflects the significant economic impact of the pandemic across the three countries,^14^ as well as the limitations of state social protection.^15^

All three states responded to the crisis through an expansion of state spending, with relief programs and emergency stimulus packages representing more than 2% of their respective GDPs (Gentilini et al. 2020a, b). Much of this, including conditional and unconditional cash transfers and expansions of social protection programmes, explicitly targeted poorer citizens and vulnerable groups.^16^ As was the case across much of the world, however, the scope and pace at which support was needed exposed limitations in targeting and state capacity, and these programs struggled to provide help for all of those affected by the pandemic (Ait Mansour 2021; Devereux 2021).

Alongside these limitations of state social protection programs, the potential role of zakat in addressing both the health and economic crises was a part of public discourse in all three countries—and across the Islamic world more broadly.^17^ This included efforts to adjust zakat practices to make them more applicable to the Covid context,^18^ as well as to mobilise contributions, both to state funds and other zakat-distributing bodies, to respond to the pandemic. The efforts to mobilise contributions through public messaging campaigns during this period shows greater urgency and attention towards zakat then generally shown by public authorities. In Pakistan, for example, state-led zakat activity during non-crisis periods remains confined to involuntary deductions on financial instruments in the month of Ramadan and no voluntary contributions are actively sought.

In Morocco, calls to contribute zakat were explicitly linked to the context of the Covid pandemic. For example, some public figures and imams called for zakat al-fitr to be donated to the Covid relief fund (Jaidani 2020). Critically, the perception of zakat’s potential role in supporting the public health and welfare response to the pandemic is intimately connected to increasing calls for the creation of a state zakat fund in Morocco. In the 2021 general election, this was not only a talking point of the National Rally of Independents led by Aziz Akhannouch, who won the most seats, but also was explicitly connected to improving funding for Morocco’s health system.

In Egypt, the foremost public religious authority, Al Azhar University, urged citizens to make zakat payments in April, three weeks before the start of the Islamic month of Ramadan, which is when such payments are usually made. Al Azhar’s House of Zakat, the largest public zakat fund in the country, explicitly framed its zakat giving in the context of the Covid pandemic, promising that funds would support the public health response.^19^ In March 2020, the grand imam of Al-Azhar, Ahmed el-Tayeb, the highest religious figure in Sunni Islam in Egypt and the administrator of its zakat fund, initiated the disbursement of $13 million in zakat payments as part of its Covid related relief efforts (Egypt Today 2020). Later in the pandemic, other religious authorities, such as the governmental Dar Al Iftah, a governmental Islamic advisory body, passed edicts allowing zakat payments to be used for the purchase of vaccines (Asharq Al-Awsat 2021).

In Pakistan, the highest governmental religious body, the Council of Islamic Ideology, explicitly made the link between zakat and Covid relief, urging citizens to make early zakat payments towards people impacted by the pandemic (Latif 2020; Lone et al. 2021). As in Egypt (Ali Sayed 2021), clerical authorities in Pakistan passed a decree in March 2020 declaring early zakat payments both permissible and desirable, given the virus’ impact on livelihoods (Latif 2020).^20^

All three states thus clearly saw zakat as an important part of the crisis response to supplement state relief efforts—and, potentially, as an opportunity to shore up the legitimacy of their leadership during the crisis and limit pressure to expand taxation. It remains unclear, however, how zakat contributors perceived state attempts to mobilise zakat during the crisis, the role of zakat in the pandemic response, and the relative importance of their obligations.

## Data

To explore citizens’ views of the role of zakat across Pakistan, Egypt, and Morocco during the pandemic, we ran nationally representative computer-assisted telephone interviews (CATI) of Sunni Muslim populations in all three countries in August–November 2020 (Appendix, Table 2).^21^ Sampling was conducted through random selection of respondents in combination with pre-specified selection criteria.^22^ To ensure a sufficient sample of zakat payers in Egypt and Morocco (where the nisab is higher), we supplemented the representative samples with surveys in each country of an additional 500 respondents who self-declared that they were eligible for zakat payment. We refer to these as the zakat-eligible sample. We capture 5484 respondents overall, of which 2648 reported to have paid zakat in the past 12 months.

Because of the limitations of phone-based surveys, we expect that our sample underrepresents lower income groups that are less likely to own phones or groups that are less likely to respond, including both women and zakat recipients. This sample bias is most evident in the context of Pakistan, where women made up only 15% of the sample (for sample descriptive statistics, see Appendix, Table 3). Where possible, we control for gender in our analysis and run robustness checks for our Pakistani sample to ensure results are not driven by gender bias. A potential under-representation of lower income groups mainly affects our discussion of zakat and state-support recipients, which we address in the analysis section below.

We also note that asking people whether they paid zakat is subject to social desirability bias, leaving respondents more likely to claim to have paid zakat when they have not than vice versa. This imposes important limitations to our analysis of overall zakat payment and highlights the need for further work in this area. Consequently, we treat the proportion of respondents who claimed to have paid zakat as an upper bound estimate. At the same time, we do not have a strong reason to believe that social desirability bias is strongly correlated with income or other demographic determinants of zakat payment, especially in a phone survey in which questions about these variables were asked after the question on payment. We therefore do not believe this potential bias affects our analysis of determinants of zakat payment. Importantly, much of our discussion is on citizen perceptions of zakat in the context of crisis, rather than personal practice, which we believe to be less affected by reporting bias.

## Citizen Perceptions of Zakat and Sadaqah During Crises

Despite state appeals to increase zakat contributions through state channels to respond to the pandemic, not all state-administered zakat funds across the globe reported increases in zakat collections during the Covid pandemic.^23^ In Pakistan, year-on-year enforced collections of zakat by the government declined by 14% in 2020. Tellingly, voluntary payments by citizens to the Central Zakat Fund also fell to around PKR 8 million (USD 50,000) during 2020, a 50% decline from the previous year and the lowest level of voluntary contributions, in nominal terms, since 2003 (State Bank of Pakistan 2020). Only considering contributions to these state funds, however, tells us little about how citizens perceive zakat in times of crisis, as only a small proportion of people pay into state-run zakat funds (where they exist) in normal times (Gallien et al. 2023).^24^

**Fig. 1:**
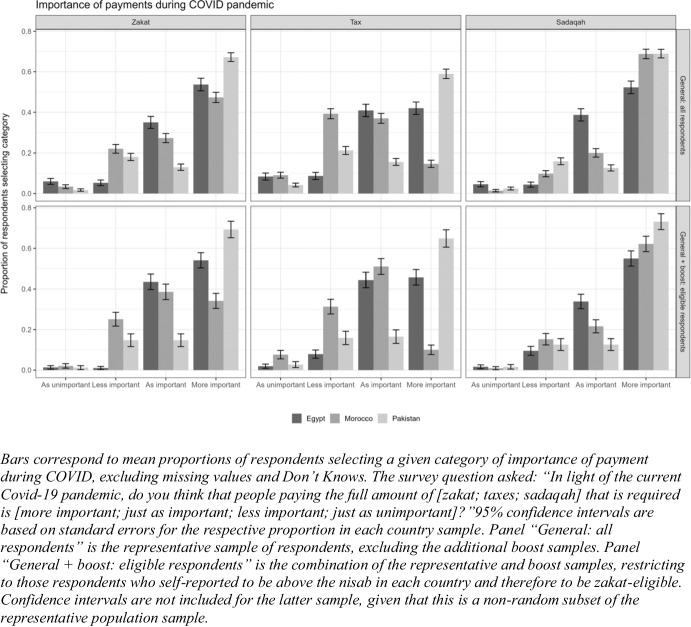
How important are zakat/tax/charity in the context of the covid pandemic?.

On the one hand, the crisis may limit willingness to contribute due to increased economic hardship, while meaning that fewer people may be eligible to pay. On the other hand, increased need and feelings of social solidarity may reinforce the importance of social protection beyond the state. As described in Fig. 1, despite economic hardship we find that people view zakat as particularly important in the context of the Covid pandemic. When asked about the degree of importance of zakat, tax, and sadaqah payments in the context of the pandemic, a majority of respondents in Egypt and Pakistan, and a high plurality in Morocco, thought the pandemic made it more important to make zakat payments.^25^ Importantly, this is true across our three country cases, despite there being different degrees of state involvement in zakat administration and different thresholds for payment, which could theoretically influence how important people view zakat overall.

**Fig. 2:**
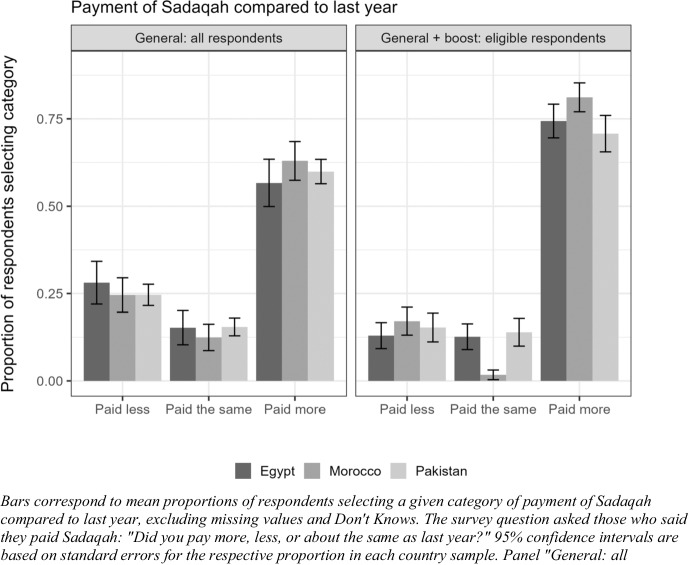
Sadaqah payment this year versus last year.

Also illustrated in Fig. 1, respondents in all three cases reported that it was more important to give sadaqah in the context of the pandemic. In line with this, a majority of individuals in all three countries reported giving more in charity in the year of the pandemic compared to the previous year (Fig. 2).^26^ Overall, this seems to suggest that rather than limiting their zakat and charity contributions in relation to personal economic hardships, citizens feel a greater sense of the importance of redistribution to those less fortunate.^27^ This in line with evidence of the power and prevalence of bottom-up responses during times of crisis, showing that, in contrast to expectations that zero-sum mentalities and conflict will emerge in times of crisis, communal solidarities often emerge or are strengthened (Dynes and Tierney 1994; Quarantelli 2008; Solnit 2010).

These findings are stark when compared to views about the role of taxation during crisis. Though tax is the financing mechanism most associated with social protection and crisis responses (e.g., Orgeira Pillai et al. 2023), a greater proportion of people believe that paying zakat and sadaqah is more important in times of crisis relative to tax payment. We also see a divergence in attitudes towards the importance of tax payments during Covid between Morocco on the one hand and Egypt and Pakistan on the other, with a higher proportion of respondents in the former reporting that it was not more important to pay tax. While we are unable to assess how different this is from a non-crisis period, we speculate that divergence is a function of citizen perceptions of the fairness of the tax system, which is low in Morocco relative to Egypt and Pakistan.^28^

## Potential Distributional Impacts of Zakat

While citizens may thus be motivated to give zakat during times of crisis, a few factors may influence its impact on equity and fairness. First, we need to consider who is more likely to pay. While zakat is often described as a means of wealth redistribution, there is little evidence of who pays in practice. There may be reason to believe, for instance, that higher income individuals may be particularly able or likely to avoid payment. This possibility was raised specifically in Pakistan in the late 1970s, with the Islamic scholar Mahmud Ahmad drawing attention to social stratification and marginalisation, which he saw as a result of “the neglect of the wealthy class in Pakistan to alleviate the situation of poor people in the country” (described in Weiss 2002, 19).

Through logistic regressions, we explore the determinants of zakat payment, finding that higher income respondents are significantly more likely to report having paid zakat relative to lower income groups (Table 1).^29^ This holds true when looking only at the subset of respondents above the eligibility threshold (col. 2), meaning that even among those for whom zakat is considered obligatory, higher income groups are more likely to report paying. Without being able to capture actual payments, we rely on self-reported zakat payment; as noted above, we thus take these reported figures as upper bound estimates of payment among higher income groups. Nevertheless, and particularly given that we don’t have any reason to believe that reporting bias varies among those that are eligible to pay, this provides some suggestive evidence that zakat is progressive in terms of who is more likely to pay.^30^ Notably, these dynamics are not unique to zakat. When we consider the determinants of respondents making charitable contributions through sadaqah in an amount equal to or higher than 2.5% of the nisab, the findings are broadly similar, especially with respect to income (see Appendix, Table 4). Without estimates of the amount paid we are unable to assess the rates of payment in relation to wealth, though given that zakat is levied at a flat rate we can surmise that it is regressive among those that pay.

**Table 1:** Determinants of zakat payment

	Determinants of payment among all respondents	Determinants of payment only among zakat-eligible respondents
High Income	1.083*** (0.096)	0.599** (0.234)
Medium Income	1.721*** (0.126)	0.856*** (0.283)
Age	− 0.174*** (0.027)	− 0.094* (0.054)
Women	0.465*** (0.085)	0.121 (0.159)
Rural Area	0.002 (0.005)	0.024*** (0.008)
Education Level	0.135*** (0.028)	0.147** (0.062)
Country FE	Yes	Yes
No. of Obs	4195	1539

Results for logistic regressions with a dummy variable indicating whether respondents had paid any zakat as the dependent variable. The survey questions used for all other parameters can be found below. High Income and Medium Income are reported against a Low Income baseline, ‘Women’ and ‘Rural Area’ are dummy variables, while Education Level is segmented into ‘low’, ‘medium’ and ‘high’. In parentheses are the respective standard errors calculated with a sandwich estimator of variance. Stars refer to: ***p < 0.01; **p < 0.05; *p < 0.1

**Fig. 3:**
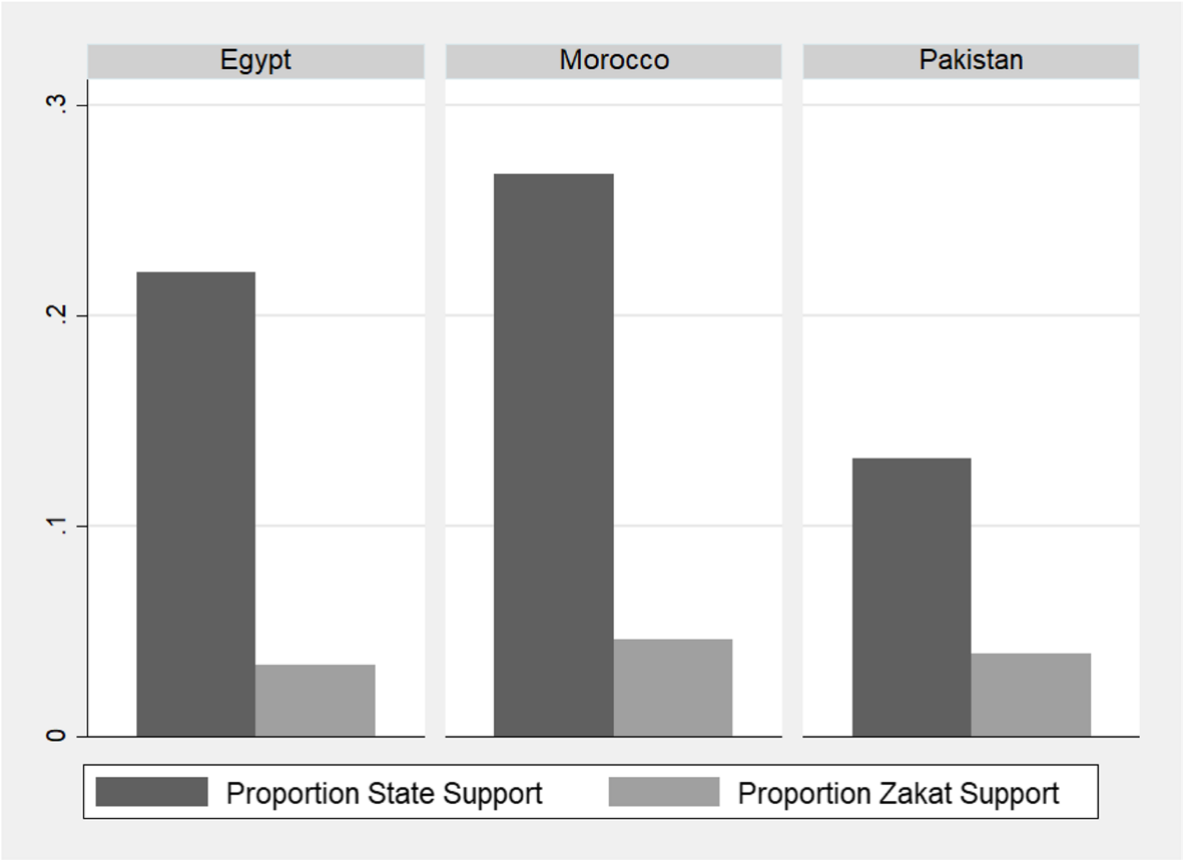
Proportion of the general sample who reported receiving zakat and state support in Pakistan, Egypt, and Morocco

We also consider who receives zakat and, in turn, whether it is likely to fill gaps left by state social protection. On the one hand, we see that few people nationally report receiving zakat; as illustrated in Fig. 3, we see that more people received state support during the Covid pandemic than received support in the form of zakat.^31^ Given the significant amount of formal state support disbursed over this period and the fact that not all of this was strictly aimed at lower income groups, this does not seem particularly surprising. Nevertheless, this data may be underestimated, given that our sample is likely to underrepresent lower income groups, as discussed above, and given that respondents may feel social stigma about receiving support (and may thus underreport). We have no reason to believe, however, that bias in our sample would affect reporting on receiving zakat more than receiving state support, and thus feel confident that the differences between the two are representative of common patterns during the crisis.

**Fig. 4:**
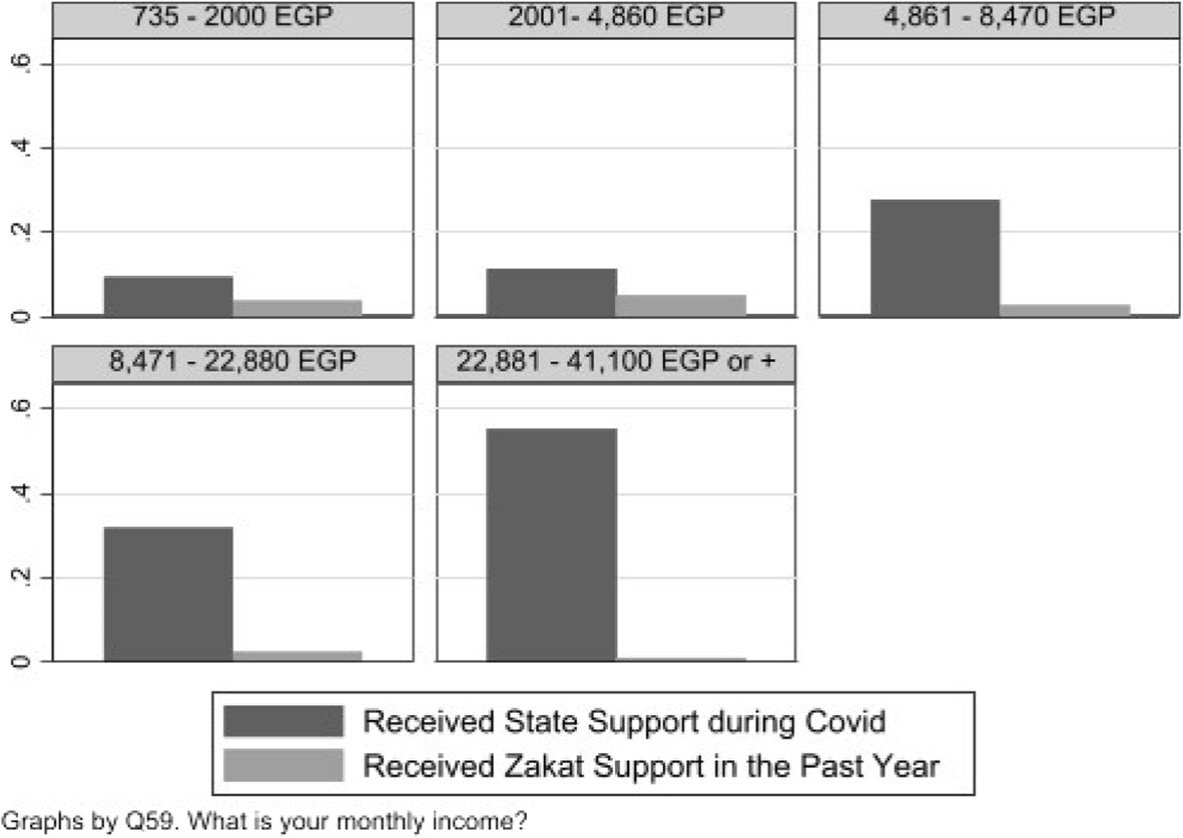
Zakat and state-relief by income distribution (Egypt)

Although our sample of zakat recipients is relatively small and our results should thus be treated with caution, we see some evidence that zakat may be effective at reaching vulnerable groups. In all three countries, we find that respondents in the lowest income groups were most likely to report having received some zakat relative to higher income groups. This may appear unremarkable, but it is worth noting that the same was not always true for state support during the pandemic. As shown in Fig. 4, higher income individuals in Egypt were *more* likely to receive state relief relative than lower income individuals.^32^ This may reflect the broader mandate of state relief during the pandemic, or the difficulties that state relief programs had in targeting lower income groups or those otherwise relatively disconnected from state structures (Centre for Economic Research in Pakistan 2021; Devereux 2021). Zakat’s apparent ability to target lower income groups may be shaped by the relatively focused definition of eligible recipients, discussed above, which contrasts with state Covid support that was not exclusively limited to marginalised groups.

Zakat’s distributional impact is likewise shaped by the channels through which people give. Our data show that the vast majority of zakat payments are made to social relations—to people within givers’ neighbourhoods, tribes, kinship groups and extended families^33^—and that people would rather give directly to people they know rather than to state funds or non-state forms of collective giving (e.g., mosques or NGOs).^34^ This is the case across all three countries, including where state funds exist (Egypt, Pakistan) and payments are enforced by the state (Pakistan).^35^

On the one hand, the fact that many people who give zakat personally know who they are giving to may mean that they have an informational advantage over the state in identifying needy recipients. This may mean that zakat is more able to reach some people that fall through the net of more institutionalised social protection programs. On the other hand, giving through social relations does not necessarily imply more equitable outcomes. Indeed, informal distribution through personal and social networks may reflect or serve to reinforce existing social exclusions and social stratification.^36^ More research is needed to study these dynamics, including whether personalized zakat giving is more likely to exclude individuals based on ethnicity, gender, or partisan affiliation.

## Conclusions

During the pandemic, the importance of social protection was clear, as were the difficulties that states face in targeting and financing social protection measures. In contexts of limited state relief, non-state social welfare fills important gaps, with zakat representing a particularly important channel for social protection beyond the state in Muslim-majority countries. While it may be plausible to think that the economic hardships induced by the pandemic may have had an impact on the scale of zakat giving, our novel data from Egypt, Morocco, and Pakistan suggest that the pandemic has not had a negative effect on the motivation to pay zakat. Instead, in the face of economic hardship, people believe it is even more important to make zakat payments in the context of the crisis.

Our exploration of the role of state action and citizen perceptions of zakat points to at least three wider implications for the political economy of zakat across Muslim-majority states and the broader role of non-state social welfare during times of crises. First, our findings speak more broadly to the importance of non-state welfare provision in times of crisis and its potential complementarities to state social protection. Such provision may fill important gaps left by state social welfare and protection, especially in instances where targeting and capacity constraints make vulnerable groups hard to reach. Given that zakat is predominately distributed within local networks, it is possible that it may be able to reach some groups that struggle with accessing state redistribution programmes, particularly where they are linked to formal employment. Despite this possibility, further research is needed to explore the extent to which decentralized zakat giving serves to address or reinforce social inequities.

Second, state mobilization of zakat during times of crisis provides a glimpse into how the state views zakat more broadly. The increasing number of state-run or state-coordinated zakat funds in recent years (Hammad 2022) may imply a perception by states that zakat can and should be more closely coordinated with other sources of public revenue (Gallien et al. 2023).^37^ While Egypt, Morocco and Pakistan all provided some form of tax relief as part of their Covid relief strategy, all explicitly sought to emphasise the importance of zakat payment in the context of the pandemic. This suggests that they view tax and zakat as quite different categories of payments in the crisis context, and perhaps even as substitutes, with zakat as a means of offloading some of the state’s responsibility when fiscal budgets are tight. The alternative, of course, would have been to focus on tax policy and administrative reform to raise revenues to support the crisis response. The fact that our case countries did not do so may point not only to the temporal disconnect—policy and administrative changes take time to lead to revenues (Gallien et al 2024)—but also to the political dynamics of taxation. It shows some limits on political will to increase taxes, at least during the early phase of the pandemic, while a desire for redistributive payments—illustrated through state encouragement of zakat—was still present. It is important to note, of course, that zakat and taxes are not actual substitutes. Whether one or the other is emphasised more in the context of a crisis has important implications for overall redistribution and equity; as well as the states’ ability to shape these. By emphasising zakat more than other means of redistribution, states forgo much of their capacity to target payments and shape redistribution and equity.

This is particularly important in the context of our third point—that there are limits to the extent to which states can take over informal institutions of public finance in a crisis context. Being able to encourage and coordinate zakat or to “tap into” zakat contributions may be considered an example of “frugal innovation”, with social welfare being provided without drawing on public resources. However, these efforts may also reflect the state’s role in “reconfiguring informal opportunities and the distribution of gains in ways that promote adverse incorporation of informal actors rather than mutual benefit” (Meagher 2018). Despite states’ efforts, our findings suggest that even in countries with state funds, the vast majority of zakat payments did not go through state-coordinated instruments during the first year of the pandemic, and in at least one case (Pakistan), voluntary contributions to state-coordinated instruments actually declined. Instead, personalized giving directly to individuals is the norm. Most zakat practice continues to exist outside of state influence on account of prevailing social perceptions around how zakat should be managed and distributed. This implies that states should rethink the role of their zakat funds as a tool of general distribution to one of specialised distribution focusing on gaps left by individual giving, which may reduce inefficiencies and maximize welfare gains.

Beyond this preliminary exploration of the role of zakat during crisis, further research is needed to fully consider zakat’s role as a tool of social welfare provision and its distributional impacts. This includes further exploration of the equity effects of zakat. While we show that economic hardship did not dampen people’s motivation to pay, we do not have more granular data that can help us understand the degree to which Covid-induced constraints shaped obligation and motivation towards zakat payments. More information is also needed on the distributional impacts, effectiveness, and targeting efficiency of zakat payments, which remain relatively unknown given the decentralized and largely undocumented nature of most payments. These underexplored points provide productive avenues for further research, which may help us better understand the role of zakat as a welfare instrument during times of crises.

## Figures and Tables

**Fig. 5:**
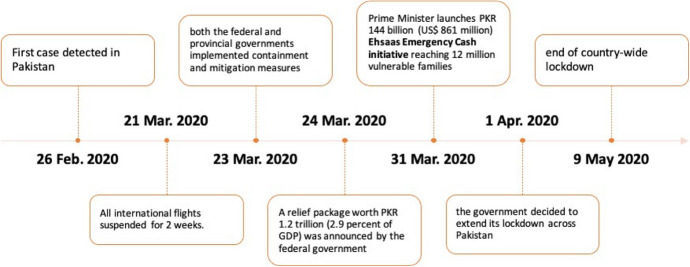
Covid timeline in Pakistan

**Fig. 6:**
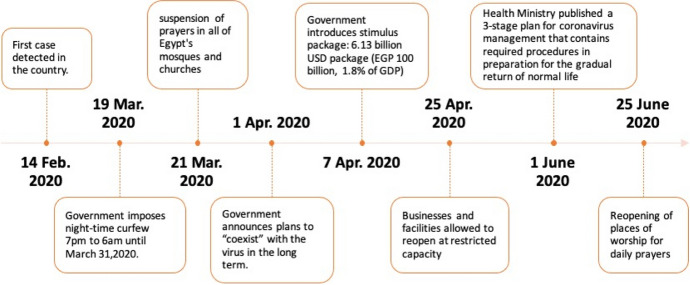
Covid timeline in Egypt

**Fig. 7:**
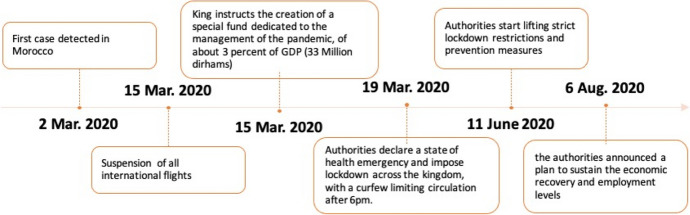
Covid timeline in Morocco

**Fig. 8:**
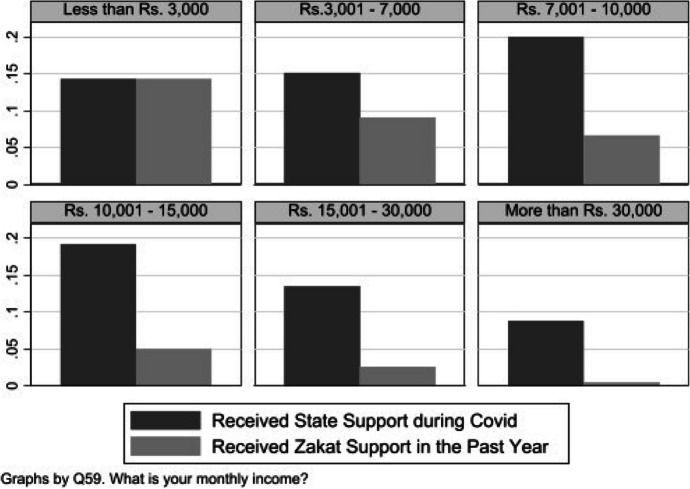
Zakat and state-relief by income distribution (Pakistan)

**Fig. 9:**
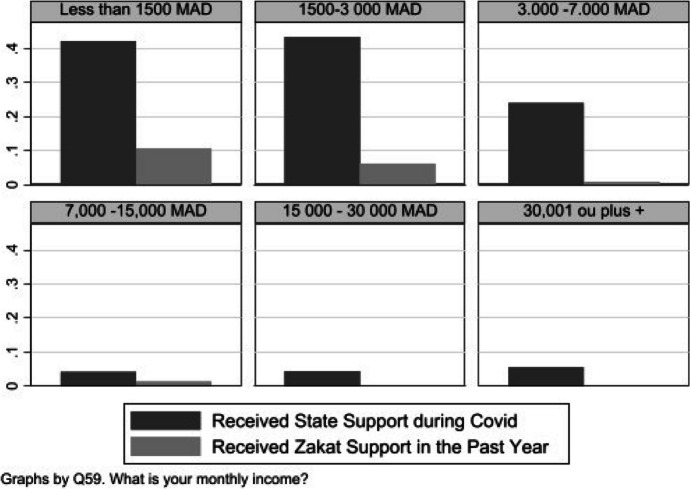
Zakat and state-relief by income distribution (Morocco)

## Data Availability

Data is available upon request.

## Appendix

**Table 2:** Sample Size

Dataset	Egypt: General	Egypt: additional eligible	Morocco: General	Morocco: additional eligible	Pakistan: General	Total
Observations	1000	500	1500	500	1984	5484
Of which reported to have paid zakat	773	445	172	425	833	2648

**Table 3:** Descriptive statistics

	*Gender*		*Income*			*Age*							*Education*					
	Women	Men	Low	Middle	High	18–24	25–34	35–44	45–54	55–64	65–74	75 +	None	Primary	Secondary	College	Postsecondary	Graduate
A. All respondents																		
Egypt	0.55	0.45	0.51	0.39	0.10	0.28	0.25	0.19	0.12	0.09	0.05	0.02	0.00	0.01	0.33	0.00	0.16	0.50
Morocco	0.51	0.49	0.47	0.43	0.10	0.23	0.22	0.21	0.16	0.10	0.06	0.02	0.05	0.11	0.17	0.22	0.34	0.11
Pakistan	0.15	0.85	0.05	0.35	0.60	0.25	0.34	0.23	0.11	0.04	0.02	0.01	0.16	0.19	0.31	0.15	0.06	0.13
Total	0.36	0.64	0.36	0.40	0.24	0.25	0.28	0.21	0.13	0.07	0.04	0.01	0.09	0.12	0.27	0.14	0.18	0.20
B. Zakat-eligible respondents																		
Egypt	0.60	0.40	0.34	0.42	0.24	0.20	0.30	0.16	0.14	0.12	0.05	0.03	0.00	0.00	0.31	0.00	0.16	0.53
Morocco	0.57	0.43	0.05	0.74	0.22	0.07	0.43	0.27	0.14	0.07	0.02	0.01	0.01	0.03	0.07	0.23	0.52	0.14
Pakistan	0.11	0.89	0.03	0.21	0.76	0.28	0.32	0.21	0.12	0.05	0.02	0.00	0.10	0.16	0.31	0.17	0.09	0.17
Total	0.45	0.55	0.17	0.51	0.32	0.18	0.35	0.21	0.14	0.08	0.03	0.02	0.03	0.06	0.23	0.13	0.27	0.29

Note: Excludes “don’t know” and missing responses

**Table 4:** Determinants of sadaqah payments over a specified threshold

	Determinants of sadaqah among all respondents	Determinants of sadaqah only among zakat-eligible respondents
High Income	1.142*** (0.097)	0.656*** (0.167)
Medium Income	1.867*** (0.012)	1.523*** (0.190)
Age	0.006 (0.028)	0.032 (0.046)
Women	0.286 (0.086)	0.108 (0.128)
Rural Area	0.510*** (0.087)	0.195 (0.161)
Education Level	0.253*** (0.028)	0.224 (0.048)
Country FE	Yes	Yes
No. of Obs	3,421	1,329

Results for logistic regressions with a dummy variable indicating whether respondents had paid sadaqah over a pre-defined amount in the past year. The amount used for each country is approximately 2.5 per cent of the nisab, or EGP 1,500, MAD 1,000, and PKR 1,600 – thereby being equivalent to the smallest mandatory zakat payment that could be obligatory in a particular context. High Income and Medium Income are reported against a Low Income baseline, ‘Women’ and ‘Rural Area’ are dummy variables, while Education Level is segmented into ‘low’, ‘medium’ and ‘high’. In parentheses are the respective standard errors calculated with a sandwich estimator of variance. Stars refer to: ****p* < 0.01; ***p* < 0.05; **p* < 0.1

## Footnotes

1 Across the world, more than 3856 new or expanded social protection initiatives were documented (Gentilini et al. 2020a).

2 There is further evidence that state-led social protection programmes are often slow to respond to crisis scenarios, face challenges in identifying and reaching vulnerable populations, are shaped by politics, and can reinforce social or gender inequities (e.g., Akerkar, Joshi, and Fordham 2016; Banerjee et al. 2022; Hickey et al. 2020; Lavers 2022; Mohamed et al. 2021; Ouma and Adésínà 2019).

3 Productive wealth refers to wealth that is accumulated for the sake of generating financial returns. It is worth nothing that throughout, the ‘year’ refers to the lunar calendar. While its specifications are unique, zakat has at times been compared with similar forms of institutionalised alms giving in other monotheistic religions, such as tithing in Christianity and maaser in Judaism require the provision of 10% of either income, profits, or agricultural yields for charitable purposes, under religious or legal obligation (Chabad n.d.; Kauppinen 2020).

4 Further bodies of literature focus on non-state actors role in providing public goods (Batley and McLoughlin 2009; van den Boogaard and Santoro 2023) or in co-producing public goods with the state (Ahlbrandt et al. 1983; Brudney 1985; Brudney and England 1983; Ferris 1984; Joshi and Moore 2004; Pammer 1992; Rich 1981; Warren, Rosentraub, and Harlow 1984; Whitaker 1980).

5 Non-state actors operating “beside the state” do so in spaces largely neglected by the state, but often with close interactions with the state; non-state actors operating “below the state” are clearly subordinate to the state; and non-state actors operating “beyond the state” may challenge state authority in sub-national arenas.

6 When referring to zakat in the survey that this paper is based on, we referred to *zakat al-mal,* the form of institutionalised almsgiving described above. This is to avoid confusion with *zakat al-fitr*, a smaller payment made at the end of Ramadan. While the latter is relevant to our discussion here as a whole, it was not the subject of our survey.

7 The categories of eligible recipients are defined as follows: “the poor and the indigent, and to those who work on [administering] it, and to those whose hearts are to be reconciled, and to [free] those in bondage, and to the debt-ridden, and for the cause of God, and to the wayfarer. [This is] an obligation from God. And God is all-knowing, all-wise.” (Quran, Al-Tawbah 9:60).

8 The most commonly cited estimate of zakat is 200 billion to 1 trillion USD (e.g., for sources that cite this 1 trillion dollar figure, see IRIN News 2012; Issa and bin Abdul Karim 2021; Rehman and Pickup 2018; Stirk 2015), though this is not well substantiated. We thus take the low estimate. Meanwhile, aid from OECD donors was USD 178.9 billion in 2021 — and this was an all-time high (OECD 2022).

9 For example, the UNHCR has operated its own zakat fund since 2019, coordinating zakat payments to support refugees in cooperation with a range of international partners.

10 Shia jurisprudence on zakat differs significantly from Sunni jurisprudence and does not consider the state to be central to zakat processes.

11 Pakistan is mainly Hanafi, Morocco is mainly Maliki, and Egypt has three main Islamic traditions: Hanafi, Maliki, and Shafi’i.

12 In 2020, this was 46,000 Pakistani Rupees (USD 284.2). Exchange rates are based on average market rate for 2020.

13 In 2020, this was set at 87,000 EGP (USD 5389.8) in Egypt and 41,000 MAD (USD 4321.4) in Morocco.

14 Pakistan’s economy contracted by 1.0% and Morocco’s by 6.3% in 2020. While Egypt did not face a recession, its reported economic growth slowed down to 3.6% in 2020 from 5.3% the preceding year. Unemployment rates rose to 6.5% in Pakistan, 7.5% in Egypt, and 11% in Morocco on account of layoffs induced by the economic slowdown as well as lockdowns and associated public health measures (World Bank Open Data n.d.).

15 For a summary and timeline of major social protection interventions in each country, see Appendix Fig. 5, Fig. 6, and Fig. 7.

16 In Pakistan, the PKR 144 billion (US$ 861 million) Ehsaas Emergency Cash program was intended to reach 12 million families across the country. Egypt expanded its conditional and unconditional cash-transfer programs, Takaful and Karama, reaching millions of new beneficiaries, while Morocco’s government used the database of its non-contributory health insurance program (RAMED) to provide almost 2.9 million households with cash transfers.

17 For example, the Islamic Development Bank (2020) explicitly called for zakat to be better coordinated with other state expenses in the context of the pandemic. See also Latif (2020).

18 Throughout Muslim-majority countries, including Egypt, Pakistan, and Morocco, governments, religious scholars and zakat organisations quickly identified the jurisprudential and technical clarifications that would be required in order to facilitate the use of zakat contributions in addressing the welfare implications of the Covid 19 pandemic. From the perspective of Islamic jurisprudence, two of the main points that required clarification was the timing of zakat contributions and the inclusion of Covid-related issues into the categories of eligible uses of zakat contributions. Both saw swift and largely supportive reactions across most Muslim majority countries. As Swandaru and Abdel (2022) point out “most sharia scholars agreed to utilize zakat for the Covid pandemic and endorse advance zakat payment.”.

19 Government hospitals also launched an appeal to support their response to Covid-19 through zakat payments, suggesting that public funding was insufficient (The New Arab 2020).

20 Additionally, as a relief measure, the provincial government in Pakistan’s most populous province, Punjab, disbursed zakat from the state-run Central Zakat Fund through mobile money (The News 2020). This is in line with adaptations seen in other Muslim countries to factor in social distancing requirements and to consider jurisprudential innovations necessary to make zakat payments as effective as possible during the pandemic (Swandaru and Abdel 2022).

21 Questionnaires available upon request. In Morocco and Egypt, the survey implementation was managed by ThinkOne, a survey firm. While the implementation team for the Moroccan survey was based in Morocco, the implementation team for the Egyptian survey was not based in Egypt. In Pakistan, the survey was implemented by Gallup Pakistan. The surveys were conducted in Arabic and Urdu, with local dialects in Egypt and Morocco being taken into account by the survey team and spot-checked by members of the research team.

22 Screening questions at the beginning of the survey were used to limit the sample to Sunni Muslim (given sizable non-Muslim minorities in Egypt and non-Sunni minorities in Pakistan) adults. Over-sampling was conducted through screening-questions (in this case, zakat eligibility).

23 While contributions reportedly increased in some countries—including Bangladesh, Ghana, India, Indonesia, Nigeria, South Africa, and the UK—they decreased in others, including Uganda and Pakistan (Hudaefi et al. 2021).

24 Reported contributions to state-run zakat funds in Pakistan and Egypt are very low. In 2020, Pakistan, only 5% of respondents reported contributing to the state fund or having zakat deducted from their bank accounts, in Egypt this number is just over 10%, much less for example than gave directly to mosques. In both countries, the vast majority of payments are made directly to social relations (Gallien et al. 2023). There is no reason to think that this pattern of giving was affected by the pandemic.

25 A *t*-test indicates that there is no statistically significant difference at a 95% confidence interval in the responses to this question based on gender in Pakistan. We discuss this in the Appendix.

26 This may be overestimated to some degree given that our sample, especially in Pakistan, captures a relatively higher income distribution than we would expect in the general population. Nevertheless, we believe this data is representative of broader trends as we expect the majority of charitable payments to be made outside of the lowest income groups, while telling us something about the giving patterns among higher income populations that are more likely to make charitable contributions. Given that gender is highly correlated with income in Pakistan, we further explore whether the results may be skewed by the fact that our sample in this context is predominately male. A *t*-test indicates that there is no statistically significant difference at a 95% confidence interval in the responses to this question based on gender in Pakistan. We discuss this further in the Appenidx.

27 This may reflect the oft-cited hadith, “No wealth (of a servant of Allah) is decreased because of charity.” Al-Tirmidhi, Hadith No. 2247.

28 In Morocco, a majority of respondents (61% in the general sample) reported that they found the amount of tax that they pay “not fair”, the lowest in a five-point scale, compared to 11% in Egypt and 5% in Pakistan.

29 Notably, despite having the same eligibility threshold, we find that more people in Egypt report paying zakat compared to Morocco (see Table 1). We hypothesise two primary drivers. The first is that despite relatively similar GDP per capita, the fact that Morocco is substantially more unequal (as evidenced by its higher GINI coefficient) has an impact on the number of people above the zakat eligibility threshold. Consequently, the proportion of the population reporting wealth above the threshold is 8% higher in Egypt than the proportion in Morocco. Second, we recognise that zakat practices are not necessarily always limited to people above the individual nisab threshold, both due to diversity in practice, differences in personal and household wealth, and differences in individual understanding of the nisab and zakat obligations. In Egypt, the proportion of people below the nisab threshold who report paying zakat is higher than in Morocco, further contributing to the difference in payment.

30 It is worth noting that women also appear to be more likely to pay on average, although this effect disappears when we control for eligibility, implying that it may be caused by an interaction between eligibility and reported income and should not be over-interpreted.

31 A *t*-test indicates that there is no statistically significant difference at a 95% confidence interval in the responses to the question about receiving state support based on gender in Pakistan. Regarding the question about receiving zakat support, there is a statistically significant gendered difference: women in Pakistan are marginally more likely to report having received zakat benefits. However, the effect is minor and does not affect the arguments on zakat and state benefits.

32 For additional graphs for Morocco and Pakistan, see Appendix, Fig. 8 and Fig. 9.

33 In all three countries, over two thirds of those who gave zakat in the past year reported giving to one of the following three categories of recipients: “Extended Family”, “Neighbours”, “Tribe and Kinship Group”. In Egypt and Pakistan, “Neighbours” represent the most common answer; in Morocco this was, “Tribe and Kinship Group”.

34 When we asked, “Do you think that it is important that people know the people they are giving zakat to, or is it enough to know that they are deserving?”, 79% of respondents in Pakistan found it either “very important” or “somewhat important” to know the zakat recipient. In Morocco (general sample), this proportion was 36% and in Egypt (general sample) it was 48%.

35 For a more detailed discussion of this, see Gallien et al. 2023.

36 This may reflect similar patterns with state-led social protection programs, which may reinforce inequities and distort the social contract depending on the nature of state capacity and pre-existing state-society relations (Alik-Lagrange et al. 2021; Lavers 2022).

37 Some evidence of this exists — Saudi Arabia, for example, extended the deadline for zakat and corporate income tax payments in 2020 (BDO 2020). However, given that the Saudi state collects zakat from corporations, this appears to be a comparative outlier.
